# Geographic Variation in Persistence of Oral Anticoagulant Treatment Among Patients with Non-Valvular Atrial Fibrillation in the United States

**DOI:** 10.3390/jcm14176265

**Published:** 2025-09-05

**Authors:** Brett D. Atwater, Risho Singh, Ali Bonakdar, Dong Cheng, Serina Deeba, Samina Dhuliawala, Michelle Zhang, Elisabeth Vodicka

**Affiliations:** 1 Division of Cardiology, Section of Electrophysiology, Inova Heart and Vascular Institute, Falls Church, VA 22040, USA; brett.atwater@inova.org; 2 Real-World Evidence, Cencora, Conshohocken, PA 19428, USA; risho.singh@cencora.com (R.S.); saminadhulz@gmail.com (S.D.); 3 US Medical Affairs, CV and Established Brands, Bristol Myers Squibb, Lawrenceville, NJ 08646, USA; dong.cheng@bms.com; 4 HTA Value & Evidence, Pfizer, New York, NY 10001, USA; serina.deeba@pfizer.com; 5 Health Economics & Outcomes Research, Bristol Myers Squibb, Lawrenceville, NJ 08648, USA; michelle.zhang@bms.com

**Keywords:** atrial fibrillation, anticoagulants, geographic variation, medication persistence, United States

## Abstract

**Background/Objectives**: Geographical variations in outcomes and oral anticoagulant (OAC) initiation among patients with nonvalvular atrial fibrillation (NVAF) in the United States (US) have been characterized; however, regional effects on OAC persistence are unknown. The study described variation in persistence with OACs among patients with NVAF across different US regions. **Methods**: The Komodo Healthcare Map was used to evaluate adult patients with NVAF, elevated stroke risk, and ≥1 pharmacy claim for an OAC between 1 January 2015 and 31 August 2022. Patients initiating treatment with an OAC (treatment-naïve) and having ≥12 months continuous enrollment were included. Persistence rates were assessed at 6, 9, 12 and 18 months among OAC- and direct OAC (DOAC)-naïve patients by 3-digit zip codes. **Results**: Of the 260,001 (Northeast = 72,507, Midwest = 59,979, South = 83,880, West = 42,778, Other/Unknown = 857) OAC-naïve patients identified, 82.2% were DOAC-naïve while 17.8% initiated warfarin. Mean follow-up time was 1101 (median = 964) and 1073 days (median = 938) in OAC and DOAC cohorts, respectively, while mean time to discontinuation was 342 (median = 190) and 329 days (median = 181), respectively. At 12 months, persistence rates ranged from 40.3% to 78.8% for OAC-naïve patients and 40.6% to 81.4% for DOAC-naïve patients. Average OAC and DOAC 12-month persistence rates were highest in the Northeast (63.5% and 63.7%, respectively) and lowest in the South (57.1% and 56.9%, respectively). **Conclusions**: Variations in 12-month persistence were consistent with existing evidence on geographic variation in NVAF-related disease burden and treatment initiation. Understanding geographic trends in prescribing patterns may provide insights into differences in treatment persistence that are relevant for clinicians seeking to address real-world barriers to care.

## 1. Introduction

Atrial fibrillation (AF) is the most prevalent type of arrhythmia, with an estimated 10.55 million cases in the United States (US) between 2005 and 2019 [1]. This number is projected to increase up to 16 million by 2050 [2]. Non-valvular AF (NVAF) accounts for over half of all AF cases and increases the adjusted risk of ischemic stroke by up to five-fold [3].

Effective stroke risk reduction in AF is dependent on adherence to guideline recommendations for initiation and persistence of oral anticoagulation (OAC) therapy. Patients with AF who initiate and then discontinue OACs are at increased risk for stroke/systemic embolism and mortality, compared to patients who initiate and persistently adhere to OAC therapy [4]. Results of a meta-analysis reported direct oral anticoagulant (DOAC) persistence ranging from 55% to 69% within the first 12 months of initiation [5]. Another study showed that the cumulative incidence of non-persistence at 12 months ranged from 43 to 59% for OACs among patients with NVAF [6]. While patients may discontinue for medical reasons (e.g., experiencing a bleeding event), non-medical factors, such as geographic disparities in access to healthcare, prescribing patterns, and social determinants of health, may also play a role [7].

Geographical differences in the prevalence of NVAF and stroke burden exist across the US, with the Southeastern “stroke-belt” experiencing higher rates of NVAF and stroke mortality [8,9]. Additionally, two studies evaluating geographic variation in OAC treatment among Medicare beneficiaries with high-risk NVAF found lower rates of OAC initiation in the South compared to the Midwest and Northeast [8,10]. Unfortunately, areas within the US that have the highest prevalence of NVAF and stroke, also have the lowest incidence of OAC initiation among potentially eligible high-risk patients. This presents a potential missed opportunity to optimize outcomes for patients in the areas that need it most [8].

While geographical variations in clinical outcomes and OAC initiation in the US have been characterized, it remains unknown whether similar differences exist in the persistence of OACs after initiation; and to our knowledge, there has been no previous nationwide analysis of geographic persistence of OACs in NVAF. The objective of this study was to describe the variation in persistence with OAC therapy among patients with NVAF at high risk of stroke across different regions of the US.

## 2. Methods

### 2.1. Data Source

This study was a real-world retrospective database analysis using closed claims from the Komodo Healthcare Map data between 1 January 2015 and 31 August 2022. The Komodo Healthcare data includes >65 billion clinical, pharmacy, and laboratory encounters for more than 320 million patients enrolled in a healthcare plan in the US from 2012 to present, >140 million of whom have closed claims from more than 150 payers with census-level representation across patient populations (e.g., age, geography, risk pools). The database is fully de-identified and compliant with the Health Insurance Portability and Accountability Act (HIPAA). Since this study did not involve the collection, use, or transmittal of individually identifiable data, it was exempt from the Institutional Review Board’s review.

### 2.2. Study Design

Patients ≥18 years of age were selected if they had an elevated risk of stroke, defined as a baseline CHA_2_DS_2_-VASc score (stands for congestive heart failure, hypertension, age ≥ 75 years (doubled), diabetes mellitus, prior stroke or transient ischemic attack (TIA) or thromboembolism (doubled), vascular disease, age 65 to 74 years, and sex category) ≥ 2 for males and ≥3 for females and at least one pharmacy prescription claim for an OAC (apixaban, dabigatran, rivaroxaban, edoxaban, or warfarin) during the identification period between 1 January 2016 to 31 August 2021. The first observed OAC claim date was designated as the index date, and the baseline period was defined as the 12 months prior to the index date. Stroke risk definition was based on the American Heart Association/American College of Cardiology (AHA/ACC) guidelines for the management of patients with AF, updated January 2019 with revised CHA_2_DS_2_-VASc scoring criteria for female sex and new clinical guidance suggesting women with a score ≥ 3 receive anticoagulants. Because the study period included dates before and after the guidelines were updated, the revised scoring guidance (CHA_2_DS_2_-VASc ≥ 3 for females) was applied to all eligible female patients regardless of the time of study entry to mitigate any impact of related treatment decisions on persistence outcomes (e.g., treatment cessation in women with CHA_2_DS_2_-VASc < 3). Patients were also required to have at least one inpatient or two outpatient claims at least 7 days apart with a diagnosis of AF in any position and within 365 days between 1 January 2015 and 31 August 2022 (study period). Patients were required to have continuous health plan enrollment with medical and pharmacy benefits for at least 12 months prior to index date (baseline period) and for at least 12 months after the index date, including the index date (follow-up period).

Patients were excluded if they had evidence of rheumatic mitral valvular heart disease (RMVHD) or any valve replacement procedures or transplant anytime during the baseline period or on the index date, venous thromboembolism or transient AF during the baseline period or on the index date, pregnancy anytime during the study period, hip- or knee-replacement surgery within 6 weeks prior to or on the index date. All patients were required to be treatment-naïve during the baseline period prior to index date; therefore, individuals were also excluded if they had evidence of more than one type of OAC on the index date or OAC treatment during the baseline period prior to index date. Among the patients meeting the selection criteria, two cohorts were established as follows: (1) patients initiating an OAC (“OAC-naïve”) and (2) a subcohort of only those patients initiating a DOAC (apixaban, dabigatran, rivaroxaban, and edoxaban) treatment (“DOAC-naïve”). The analysis was conducted among both cohorts.

**Figure 1:**
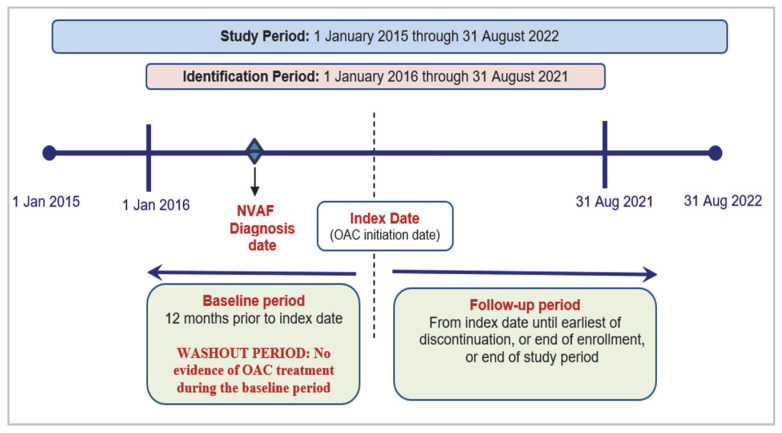
Study design. Key: OAC–oral anticoagulant; NVAF–non-valvular atrial fibrillation.

Baseline demographic and clinical characteristics were evaluated using all available data within the 12-month baseline period up to and including index date. Patients’ data were assessed from the index date censored in the follow-up period for the following criteria: discontinuation of OAC, disenrollment or end of study period. Figure 1 provides a visual representation of the study design for OAC-naïve patients with NVAF at high risk of stroke.

Patients with only one prescription claim (index claim) with missing days of supply, and patients with ≥2 prescription claims with missing days of supply on all the claims were excluded. Patients with missing days of supply only on the index date were imputed with mode of non-missing days of supply. For patients with missing days of supply on the non-index claims, imputation with mode was carried out as long as 50% of the claims had non-missing days of supply.

### 2.3. Outcome Measures

The proportion of patients who discontinued treatment during the follow-up period was evaluated in both OAC- and DOAC-naïve cohorts, with discontinuation defined as no evidence of an OAC prescription for ≥60 days from the last day of supply of the last-filled prescription during the follow-up period, and the discontinuation date defined as the date of the last day of supply of the last-filled prescription. The 60-day permissible gap aligns with the ranges used in previous OAC studies of persistence [5]. For OAC- and DOAC-treated cohorts, as long as there was a claim for any DOAC within the 60-day gap, the patients were considered to be in continuation of treatment. The proportions of patients persistent at 6, 9, 12, and 18 months were also evaluated, with persistence defined as the number of days from treatment initiation (index date) to treatment discontinuation for the specified period.

To evaluate 12-month persistence rates among the OAC- and DOAC-naïve patients with NVAF at high risk of stroke by geography, the persistence rates were calculated for each patient at the 3-digit zip code level. Static heatmaps were then generated to show geographic variation in the 12-month persistence rates across the 50 US states, excluding the territories of Puerto Rico, Guam, and the Virgin Islands, at the 3-digit zip code level. The 3-digit zip codes with fewer than 50 patients were excluded from the heatmaps. This threshold was established to reduce potential biases from small sample sizes while maximizing the number of included zip codes. While lower thresholds were also evaluated, a minimum sample size of 50 individuals ensured representation from all states, allowed for inclusion of the majority (75%) of 3-digit zip code areas, and addressed issues related to unstable estimates, and has been previously applied for nationally representative geographic-level research [11].

The distribution of 30- and 90-day supply prescriptions across different US geographic regions for OAC- and DOAC-naïve patients with >1 prescription claims were evaluated through the follow-up period. The proportion of claims for each cohort by region was reported relative to the total number of claims. Analyses of claims per patient by geographic region among OAC- and DOAC-naïve patients with >1 prescription claim (including index claims), as well as for patients who were persistent at 12 months, were conducted.

A sensitivity analysis was conducted to evaluate persistence at 6, 9, 12, and 18 months when a shorter discontinuation gap was considered to define discontinuation. For the sensitivity analyses, discontinuation was defined as no evidence of an oral anticoagulant prescription for ≥30 days (vs. ≥60 days in the main analysis) from the last day of supply of the last-filled prescription in the follow-up period. Persistence was evaluated using a ≥30-day discontinuation gap, enabling analysis of how a shorter gap period impacts non-persistence outcomes [6,12].

### 2.4. Statistical Analysis

Descriptive statistics (i.e., frequency, percent, mean, standard deviation, median, 25th percentile [Q1], 75th percentile [Q3], and interquartile range [IQR] reported as Q1–Q3) were used to describe baseline demographic and clinical characteristics, and outcomes during the follow-up period.

All data management and statistical analysis programs were created using SAS 9.4 (SAS Institute Inc., Cary, NC, USA) and in compliance with standard operating procedures (SOPs) related to data management and analysis. The study was conducted in accordance with the International Society for Pharmacoepidemiology Guidelines for Good Pharmacoepidemiology Practices [13].

## 3. Results

**Figure 2:**
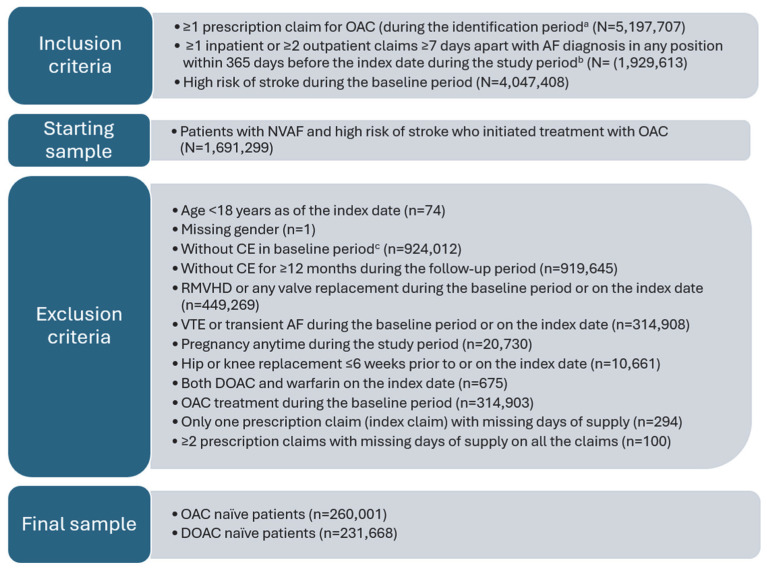
Patient selection criteria. ^a^ OAC included apixaban, dabigatran, rivaroxaban, edoxaban, or warfarin; identification period was from 1 January 2016 to 31 August 2021. ^b^ Study period was from 1 January 2015 to 31 August 2022. ^c^ Baseline period included time frame of 12 months prior to index date. AF–atrial fibrillation; CE–continuous enrollment; DOAC–direct oral anticoagulant; NVAF–non-valvular atrial fibrillation; OAC–oral anticoagulant; RMVHD–rheumatic mitral valvular heart disease; VTE–venous thromboembolism.

After applying the patient inclusion and exclusion criteria, 260,001 patients were identified with at least 12 months of continuous enrollment (CE) with medical and pharmacy benefits and had evidence of an NVAF diagnosis that had newly initiated treatment with an OAC (“OAC-naïve”) (Figure 2), of which almost 213,668 (82.2%) patients had newly initiated treatment with a DOAC (“DOAC-naïve”). Among the 260,001 patients in the OAC-naïve cohort, 228,878 (88.0%) patients had at least 18 months CE with medical and pharmacy benefits. Among 213,668 patients in the DOAC-naïve cohort, 188,453 (88.2%) had at least 18 months CE.

### 3.1. Baseline Characteristics

In general, baseline characteristics among OAC- and DOAC-naïve patients with NVAF were similar. Baseline demographic and clinical characteristics of OAC- and DOAC-naïve patients are presented in Table 1.

The mean ages of OAC- and DOAC-naïve patients were 71.8 and 71.3 years, respectively, with proportionately more male than female patients (53.7% and 52.8% male, respectively, in the OAC- and DOAC-naïve cohorts). Patients with high-risk of stroke in both cohorts had a mean Charlson Comorbidity Index (CCI) of 3.0, with nearly 50% of patients having a mean CCI score of 3 or greater. The mean baseline CHA_2_DS_2_-VASc score was 3.7 in both groups, while the median score was 4.0 and 3.0 in the OAC- and DOAC-naïve patients, respectively. For other relevant baseline demographic and clinical characteristics, refer to Table S1 in the Supplementary Material.

### 3.2. Discontinuation and Persistence Among Patients with NVAF at High Risk of Stroke During the Follow-Up Period

Results for follow-up time, time to discontinuation, and persistence are provided in Table 2. The mean follow-up time in the OAC-naïve and DOAC-naïve cohorts was 1101 days (median = 964 days) and 1073 days (median = 938 days), respectively. Among OAC-naïve patients, 63.5% of patients discontinued treatment during the follow-up period and the mean time to treatment discontinuation was 342 days (median = 190 days [IQR: 61–463 days]). Among DOAC-naïve patients, 62.4% of patients discontinued treatment during the follow-up period, and the mean time to treatment discontinuation was 329 days (median = 181 days [IQR: 59–445 days]).

When the follow-up was restricted to 12 months in the OAC-naïve cohort, 39.8% patients discontinued treatment with a mean time to treatment discontinuation of 109 days (median = 89 days [IQR: 29–178 days]). Similarly, 40.0% of DOAC-naïve patients discontinued treatment and the mean time to treatment discontinuation was 107 days (median = 89 days [IQR: 29–174 days]).

Among patients with 12 months of follow-up, approximately 69%, 62%, and 60% of patients in both OAC- and DOAC-naïve cohorts were found to be persistent at 6 months, 9 months, and 12 months, respectively. In the subset of patients that had at least 18 months of follow-up, approximately 50% of patients in the OAC-naïve and 49% of patients in the DOAC-naïve cohorts were persistent with treatment at 18 months.

**Figure 3:**
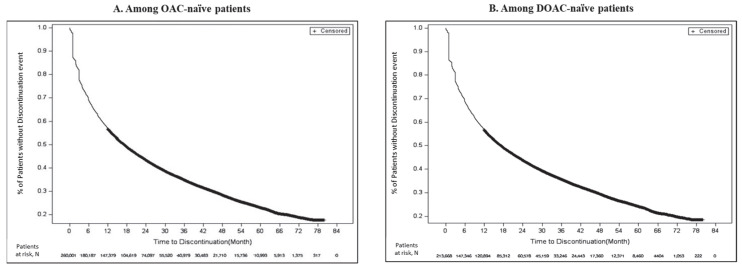
Kaplan–Meier survival curves for time to discontinuation among OAC- and DOAC-naïve patients.

Kaplan–Meier survival curves for time to discontinuation as defined by a 60-day gap among OAC- and DOAC-naïve patients throughout the entire follow-up period is presented in Figure 3.

Kaplan–Meier survival curves from sensitivity analyses assessing time to discontinuation as defined by a 30-day gap is presented in Figure S1 in the Supplementary Material.

Table S2 in the Supplementary Material reports discontinuation and persistence when discontinuation was defined as no evidence of an oral anticoagulant prescription for ≥30 days from the last day of supply of the last-filled prescription in the follow-up period. In general, using a shorter time frame for discontinuation resulted in a decrease in persistence rates of approximately 10% compared with those observed with the primary analysis (discontinuation was defined as ≥60-day gap).

#### 3.2.1. Geographic Variation in Treatment Persistence

**Figure 4:**
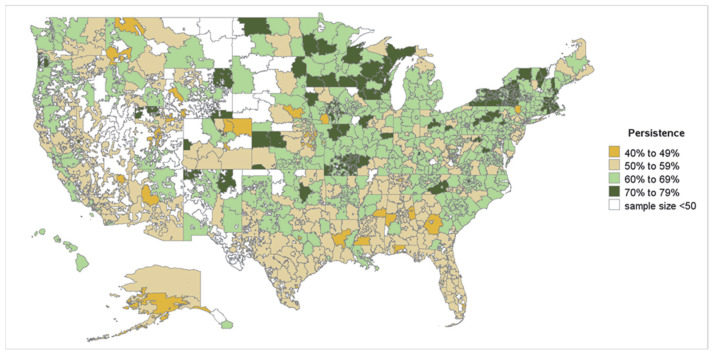
Geographic variation in 12-month persistence among OAC-naïve patients with NVAF by 3-digit zip code. OAC—oral anticoagulant; NVAF—nonvalvular atrial fibrillation. Note: For the above heatmap, 3-digit zip codes with at least 50 patients were considered. Heatmaps were generated for the 50 US states and excluded the territories of Puerto Rico, Guam, and Virgin Islands. White shade represents unpopulated areas, 3-digit zip codes with <50 patients, and 3-digit zip codes with missing information on treatment.

**Figure 5:**
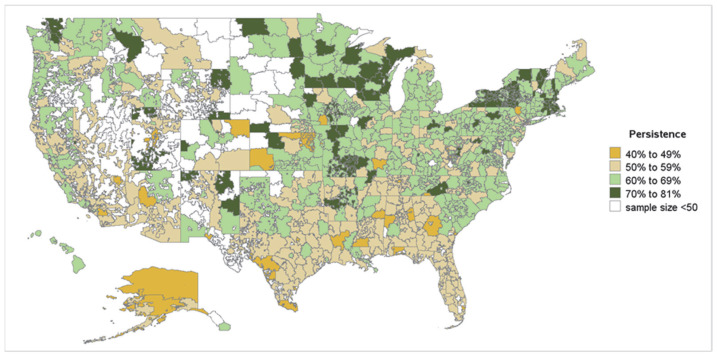
Geographic variation in 12-month persistence among DOAC-naïve patients with NVAF by 3-digit zip code. DOAC—direct oral anticoagulant; NVAF—nonvalvular atrial fibrillation. Note: For the above heatmap, 3-digit zip codes with at least 50 patients were considered. Heatmaps were generated for the 50 US states and excluded the territories of Puerto Rico, Guam, and Virgin Islands. White shade represents unpopulated areas, with 3-digit zip codes with <50 patients, and 3-digit zip codes with missing information on treatment.

In the zip code-level analysis for the OAC- and DOAC-naïve cohorts, 659 and 623 3-digit zip codes were included, respectively. Among included zip codes, the 12-month persistence rates ranged from 40.3% to 78.8% for OAC-naïve patients and 40.6% to 81.4% for DOAC-naïve patients. In general, the 12-month persistence rates for both OAC- and DOAC-naïve populations were observed to be higher in 3-digit zip codes located in the Northeast and Midwest regions of the US, while 3-digit zip codes in the South displayed lower rates, with some variation noted (Figure 4 and Figure 5). OAC- and DOAC-naïve patients residing in zip codes within the stroke-belt had lower mean persistence (58.3% and 58.4%, respectively) than OAC- and DOAC-naïve patients in zip codes outside the stroke belt (61.3% and 61.0%, respectively).

**Figure 6:**
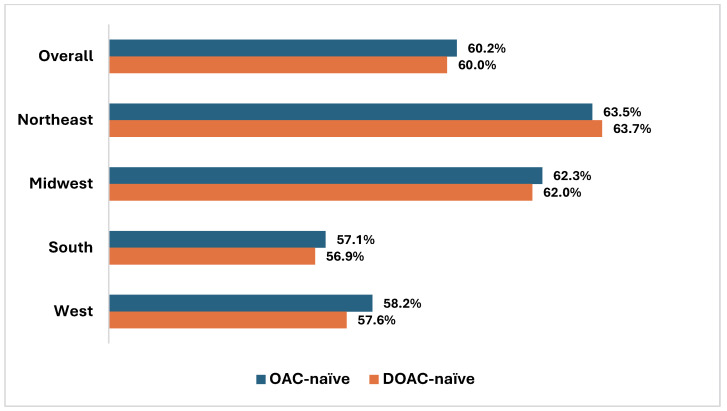
Twelve-month persistence rates for OAC- and DOAC-naïve patients by US geographic region. Key: DOAC—direct oral anticoagulant; OAC—oral anticoagulant. Note: “Overall” includes Northeast, Midwest, South, West, other, and unknown regions. “Other” includes Puerto Rico, Guam, and Virgin Islands.

At the regional level (Northeast, Midwest, South, West), average persistence was lowest in the South, with approximately 57% of OAC- and DOAC-naïve patients still persistent at 12 months, compared to 64% in the Northeast where an average of 12-month persistence was highest (Figure 6).

#### 3.2.2. Geographic Variation in Persistence Stratified by Days’ Supply

The average number of claims per patient and days’ supply prescribed by geographic region among overall and persistent OAC- and DOAC-naïve patients with NVAF with >1 prescription claim, including index claims, are reported in Table S3 in the Supplementary Material. Among 12-month persistent OAC-naïve patients, the highest average number of claims per patient for a 30-day supply was found in the South (6.0) and the lowest in the Midwest (5.5) (Table S3); for a 90-day supply, the average numbers of claims per persistent patient were similar across US regions (approximately 2). Among persistent DOAC-naïve patients, the average numbers of claims per patient for a 30-day supply were similar among US regions, with the highest numbers observed in the South and West (6.3 in both regions), and lowest was observed in the Midwest (6.0); for a 90-day supply, mean number of claims per patient were similar across US regions (2.0–2.2) (Table S3). The distribution of 30- and 90-days’ supply by geographic region when excluding index claims is provided in Table S4 in the Supplementary Material.

## 4. Discussion

Among both OAC- and DOAC-naïve populations, the mean overall persistence at 12 months was approximately 60%. This is similar to estimates in the published literature. For example, a meta-analysis of 36 studies reported a pooled 12-month persistence rate of 62% (95% confidence interval [CI]: 56–68%), of which almost 60% had a permissible gap of 56–60 days between fills to define persistence [5]. In another study pooling data from five large national claims databases, the cumulative incidence of non-persistence at 12 months ranged from 42.7% to 58.9% for patients receiving apixaban, dabigatran, rivaroxaban, or warfarin, slightly lower than overall persistence in the current study [6].

At the 3-digit zip code level, mean persistence ranged approximately 40% to 80% in both cohorts. When aggregated to the regional level (Northeast, South, Midwest, West), differences in persistence rates by geography were identified. Specifically, higher 12-month persistence in OAC- and DOAC-naïve patients with NVAF was observed in the Northeast (~64%) while lower 12-month persistence was observed in the South (~57%). Multiple factors may influence regional variations in OAC and DOAC treatment persistence among patients with NVAF, such as medical, financial, and educational factors and access to ambulatory healthcare resources. In addition, the study observation period overlapped with the COVID-19 pandemic, which may have affected treatment persistence rates [14].

The findings of the current study of lower persistence rates in the South are congruent with geographic variation previously identified for OAC and DOAC initiation. Geographic patterns of stroke deaths in the US are well documented for both black and white adults, with concentrations of high stroke death rates consistently reported in Southeastern states (i.e., North Carolina, South Carolina, Georgia, Tennessee, Alabama, Mississippi and Arkansas, and Louisiana), a region commonly referred to as the “stroke belt” [15]. Previous studies have observed higher stroke rates, systemic embolic event rates, and excess stroke mortality, but lower overall frequency of OAC initiation in the Southeastern US [8,9,16]. Results of a real-world retrospective analysis of claims data for Medicare patients with NVAF found that the South had the lowest rates of OAC initiation, the highest clinical risk scores, and the highest incidence of stroke/systemic embolism and bleeding compared with other regions of the US [8]. This study shows that patients in these high- risk areas are also less likely to persistently adhere to OAC and DOAC therapy after initiation. The lack of initiation and persistent utilization of OAC for prevention of stroke in areas with high AF prevalence may explain the higher observed incidence of stroke and systemic embolism in the Southern US.

Geographic density and proximity to healthcare services may also play a role. Results of the 2020 US census found that the South and Midwest continue to have higher proportions of their population living in rural areas (~25%) compared to Northeast (~16%) and West (~11%) [17]. Compared to urban areas, stroke mortality is approximately 30% higher in rural areas, where stroke incidence is also higher (odds ratios [ORs], 1.23; 95% CI, 1.01–1.51 and 1.30; 95% CI, 1.03–1.62, in large rural town/cities and small rural towns or isolated areas, respectively, relative to urban areas) [16,18]. The existence of lower OAC and DOAC persistence rates in the South may likewise be influenced by the greater proportion of individuals in Southern states living in rural areas. This study did not evaluate treatment patterns stratified by rural vs. urban settings or other indicators of access to care, such as density of health services. Future research will evaluate the relationship between rurality, access to care, and persistent use of OAC and DOAC therapy.

In a study involving Medicare beneficiaries with new initiation of OAC for NVAF with high stroke risk, lower DOAC use was observed in the Midwest while DOAC was most frequently chosen as the OAC to initiate in 3-digit zip codes located in the South [8]. In another retrospective study among commercially insured patients with NVAF at high risk of stroke, the overall prevalence rate of DOAC treatment was 85%, with the highest rate observed in the Northeast followed by Midwest and the lowest rate observed in the West followed by the South [19].

Understanding geographic trends in prescribing patterns may provide valuable insights into differences seen in treatment persistence across regions and highlight opportunities for addressing barriers to medication persistence for patients. In general, patients who are prescribed longer days of supply prescriptions tend to have greater persistence than those who had shorter days of supply prescriptions [20]. However, data from the US Centers for Disease Control and Prevention indicate significant differences in prescribing patterns between states and regions, particularly in terms of days of supply [20]. The Northeast tends to have longer prescriptions compared to Southern states that often have shorter prescriptions. Similarly, this study also observed a higher rate of 30-day OAC supply in the South. These trends can partly be attributed to regional differences in healthcare systems and insurance coverage. Southern states typically have more restrictions on Medicaid and lower rates of insurance coverage, which may lead to shorter days of supply for prescriptions and higher out-of-pocket costs for patients. On the other hand, states in the Northeast and West may have policies that encourage longer days of supply for prescriptions, often with a focus on reducing the frequency of pharmacy visits and improving adherence [20].

Regional differences in clinical decision-making may also impact treatment persistence. The American Heart Association and European Society of Cardiology guidelines recommend use of DOACs for AF patients at high risk for stroke. However, studies demonstrate key differences between primary care physicians’ (PCPs) and cardiologists’ knowledge, practices, and attitudes on anticoagulation for NVAF. In a study conducted by interviewing PCPs or cardiologists, 24.3% of PCPs and only 1.2% of cardiologists never used CHA_2_DS_2_-VASc risk stratification when assessing the risk of stroke and bleeding among patients with NVAF before treating [21]. The study inferred that when selecting anticoagulation for NVAF, PCPs had greater concern with the anticoagulant’s reversibility, while cardiologists were more concerned with the risk of stroke. This study also found that cardiologists were more likely than PCPs to prescribe DOACs; however, to our knowledge, studies have not evaluated regional variation in clinician knowledge, attitude, and practice related to NVAF treatment. This evidence may provide insights into ways to reduce disparities in patient care.

The current study is unique in representing geographical variations in OAC and DOAC persistence among patients with NVAF at high risk of stroke immediately after treatment initiation. This study adds to the existing literature on real-world use of OACs and DOACs by characterizing overall treatment persistence and differences by geographic region through analysis of a large claims database. While geographic variation appears to play a role in persistence to OACs and DOACs, intersectional factors may impact access to care, adherence, and persistence to treatment. Patient-related factors (e.g., demographics, medical conditions, behavioral factors, patient education, insurance type), prescriber-related knowledge (e.g., adherence to treatment guidelines, awareness of recommendations, 30- vs. 90-day fills, frequency of patient follow up), and healthcare system-related factors (e.g., structure of healthcare system, health plan policies, technology tools support, out-of-pocket costs of care) also influence adherence and persistence to OACs [22,23]. These may represent areas of future research, which include fully identifying predictors of low rates of OAC use, analyzing adherence and persistence to treatment, and identifying opportunities for reducing barriers to optimal patient utilization.

There are several limitations of this study. First, although claims data are commonly used for real-world studies, there might be a lack in medical accuracy and completeness due to the administrative nature of the data. For example, as treatment discontinuation in this study was evaluated based on prescription fill data, the precise date for a patient’s discontinuation cannot be determined. Similarly, treatment persistence does not imply adherence, as the presence of a claim for a filled prescription does not indicate whether the medication was consumed or taken as prescribed. Additionally, prescription samples provided to patients cannot be captured in claims data; therefore, persistence may be underrepresented in this study. However, in absence of more granular data, leveraging available administrative claims data on prescription fills is a widely used approach for assessing trends in patient treatment patterns and identifying patient populations at greater risk of non-persistence and medication abandonment [24,25]. Also, the imputation of missing days’ supply may have introduced bias and misclassification, as it relies on assumptions that may not reflect real-world patient behaviors which may have affected the persistence analyses.

Also, the geographic distribution of the AF commercial patient population in Komodo may not be generalizable to other populations. Komodo data are typically considered generalizable to the US population based on sourcing from diverse payers (i.e., over 150 complete datasets) and similar distribution in terms of geography and patient demographics when compared to the US population as reported in the National Health Interview Survey in 2019. However, the database predominantly includes patients with commercial insurance (including Medicare Advantage), and patients with Medicare fee-for-service and Medicaid were not represented in the study, which limits the generalizability of the results in these populations. Another limitation is that, although the study did not examine geographic variations by race or ethnicity, the lack of race and ethnicity data may have influenced the observed persistence rates across regions. Additionally, factors that may correlate with both geographic variation and treatment persistence, such as socioeconomic characteristics, were not evaluated. Finally, this is a descriptive study to examine the geographic variation in OAC treatment persistence; and therefore, associations between any factors and persistence were not evaluated. Persistence rates might be driven by medical or non-medical reasons such as out-of-pocket costs, formulary changes, insurance changes, physician preferences, and other reasons affecting healthcare access; however, the study did not capture these potential contributors to treatment discontinuation. Given the nature of the study, investigating potential confounding by measured or unmeasured confounders was not within the scope or primary interest of the analysis, and the study focused solely on observing persistence by geographical region.

## 5. Conclusions

In this real-world retrospective database analysis, higher 12-month persistence in OAC- and DOAC-naïve patients with NVAF was observed in the Northeast region, and lower 12-month persistence was observed in the South region of the US, demonstrating consistent trends with existing evidence on geographic variation in NVAF-related disease burden and treatment initiation. Understanding geographic trends in prescribing patterns may provide valuable insights into differences seen in treatment persistence across regions and highlight opportunities for addressing barriers to medication persistence for patients. To conclude, this study demonstrates notable geographic variation in oral anticoagulant persistence, emphasizing the importance of addressing regional barriers and informing future research and policies to improve equitable access and adherence to treatment.

## Figures and Tables

**Table 1:** Baseline demographic and clinical characteristics among OAC- and DOAC-naïve patients with NVAF at high risk of stroke.

Characteristic	OAC-Naïve Population N = 260,001 (100%)	DOAC-Naïve Population ^a^ N = 213,668 (100%)
**Demographic characteristics (measured as of index date)**		
Age, years		
Mean, SD	71.8 (10.6)	71.3 (10.7)
Median (Q1–Q3)	73 (64–81)	72 (64–80)
Gender, n (%)		
Male	139,727 (53.7%)	112,763 (52.8%)
Female	120,274 (46.3%)	100,905 (47.2%)
Race, n (%)		
White	188,694 (72.6%)	152,106 (71.2%)
Black or African American	21,474 (8.3%)	17,880 (8.4%)
Other race ^b^	12,824 (4.9%)	11,027 (5.2%)
Missing	37,009 (14.2%)	32,655 (15.3%)
Ethnicity, n (%)		
Hispanic or Latino	22,180 (8.5%)	18,787 (8.8%)
Not Hispanic/not Latino	112,746 (43.4%)	92,937 (43.5%)
Missing/unknown	125,075 (48.1%)	101,944 (47.7%)
Geographic region, n (%)		
Northeast	72,507 (27.9%)	59,723 (28.0%)
Midwest	59,979 (23.1%)	46,945 (22.0%)
South	83,880 (32.3%)	71,614 (33.5%)
West	42,778 (16.5%)	34,632 (16.2%)
Other ^c^	649 (0.2%)	597 (0.3%)
Unknown	208 (0.1%)	157 (0.1%)
**Clinical characteristics (measured during baseline period)**		
CCI		
Mean (SD)	3.0 (2.5)	3.0 (2.5)
Median (Q1–Q3)	2 (1–4)	2 (1–4)
CHA_2_DS_2_-VASc score		
Mean (SD)	3.7 (1.4)	3.7 (1.4)
Median (Q1–Q3)	4 (3–5)	3 (3–5)
HAS-BLED score		
Mean (SD)	3.5 (1.1)	3.5 (1.1)
Median (Q1–Q3)	3 (3–4)	3 (3–4)
Baseline comorbidities (top five), n (%)		
Hypertension	236,151 (90.8%)	194,800 (91.2%)
Diabetes	110,785 (42.6%)	90,207 (42.2%)
Coronary artery disease	110,570 (42.5%)	89,527 (41.9%)
Congestive heart failure	85,003 (32.7%)	68,440 (32.0%)
Anemia and coagulation defects	77,318 (29.7%)	61,825 (28.9%)
Baseline medications use (top five), n (%)		
Inhibitors of warfarin ^d^	168,223 (64.7%)	143,165 (67.0%)
Renin-angiotensin system antagonist	137,091 (52.7%)	117,381 (54.9%)
Statin	130,208 (50.1%)	110,932 (51.9%)
Beta blocker	114,364 (44.0%)	97,484 (45.6%)
Diuretic	109,566 (42.1%)	91,894 (43.0%)

ACE—angiotensin-converting enzyme; ARB—angiotensin receptor blocker; CCI—Charlson Comorbidity index; DOAC—direct oral anticoagulant; NVAF—nonvalvular atrial fibrillation; OAC—oral anticoagulant; Q1—25th percentile; Q3—75th percentile; SD—standard deviation; SE—systemic embolism. ^a^ The DOAC-naïve population with NVAF represents a subcohort of the OAC-naïve population with NVAF. ^b^ Includes Asian or Pacific Islander, Alaskan Native or Native American, and other race categories. ^c^ Includes Puerto Rico, Guam, and Virgin Islands. ^d^ Includes therapies classified as CYP2C9 inhibitors, CYP1A2 inhibitors, and CYP3A4 inhibitors.

**Table 2:** Outcomes for OAC- and DOAC-naïve patients with NVAF at time of follow up.

Characteristic	OAC-Naïve Population	DOAC-Naïve Population
Sample size (12-month follow-up), n (%)	260,001 (100.0%)	213,668 (100.0%)
Sample size (18-month follow-up ^a^), n (%)	228,878 (88.0%)	72 (64–80)
Measured over the entire follow-up period		
Follow-up time, days		
Mean (SD)	1101 (563)	1073 (549)
Median (Q1–Q3)	964 (615–1527)	938 (602–1462)
**Discontinuation during entire follow-up period**		
Proportion of discontinuers, n (%)	165,209 (63.5%)	133,433 (62.4%)
Time to discontinuation with 60-day gap, days		
Mean (SD)	342 (395)	329 (387)
Median (Q1–Q3)	190 (61–463)	181 (59–445)
**Time to discontinuation during 12-month follow-up period**		
Proportion of discontinuers among those with up to 12 months follow-up, n (%)	103,355 (39.8%)	85,425 (40.0%)
Time to discontinuation with 60-day gap, days		
Mean (SD)	109 (84)	107 (84)
Median (Q1–Q3)	89 (29–178)	89 (29–174)
**Persistence among patients with up to 12 months of follow up**		
Proportion of patients, n (%)		
Persistent at 6 months	180,187 (69.3%)	147,346 (69.0%)
Persistent at 9 months	161,890 (62.3%)	132,502 (62.0%)
Persistent at 12 months	156,646 (60.2%)	128,243 (60.0%)
**Persistence among a subset of patients with up to 18 months of follow up**		
Proportion of patients, n (%)		
Persistent at 18 months ^a^	113,770 (49.7%)	93,180 (49.4%)

DOAC—direct oral anticoagulant; NVAF—nonvalvular atrial fibrillation; OAC—oral anticoagulant; Q1—25th percentile; Q3—75th percentile; SD—standard deviation. ^a^ Persistence at 18 months (exploratory outcome) is calculated among those patients who had 18 months of continuous enrollment during the follow up period. The denominator for the 18-month persistence calculation is a subset of patients with 12 months follow-up period.

## Data Availability

The original contributions presented in this study are included in the article/Supplementary Material. Further inquiries can be directed to the corresponding authors.

## Supplementary Materials

The following supporting information can be downloaded at: https://www.mdpi.com/article/10.3390/jcm14176265/s1, Figure S1: Kaplan–Meier survival curves for time to discontinuation (by 30-day gap) among OAC-naïve and DOAC-naïve patients—Sensitivity analysis; Table S1: Other baseline demographic and clinical characteristics among OAC- and DOAC-naïve patients with NVAF at high risk of stroke; Table S2: Outcomes for OAC- and DOAC-naïve patients with NVAF at time of follow up after reducing treatment gap from ≥60 to ≥30 days to define discontinuation (sensitivity analyses); Table S3: Mean number of claims for all OAC-naïve and DOAC-naïve patients with NVAF by region and days’ supply; Table S4: Distribution of 30- and 90-day supply among OAC-naïve and DOAC-naïve patients with NVAF with >1 prescription claims by geographic region.

## Abbreviations

The following abbreviations are used in this manuscript:

ACC	American College of Cardiology
ACE	Angiotensin-converting enzyme
AHA	American Heart Association
AF	Atrial fibrillation
ARB	Angiotensin receptor blocker
CCI	Charlson Comorbidity Index
CE	Continuous enrollment
CI	Confidence interval
COVID	Coronavirus disease
DOAC	Direct oral anticoagulant
HIPAA	Health Insurance Portability and Accountability Act
IQR	Interquartile range
NC	North Carolina
NVAF	Non-valvular atrial fibrillation
OAC	Oral anticoagulation
OR	odds ratio
PCP	primary care physician
Q1	25th percentile
Q3	75th percentile
RMVHD	Rheumatic mitral valvular heart disease
SD	standard deviation
SE	systemic embolism
SOP	standard operating procedure
US	United States
VTE	Venous thromboembolism
