# Supplementary material for: Geographic Variation in Persistence of Oral Anticoagulant Treatment Among Patients with Non-Valvular Atrial Fibrillation in the United States
Source: J Clin Med. 2025 Sep 5;14(17):6265. doi: 10.3390/jcm14176265 (PMC12429831; doi:10.3390/jcm14176265)
Supplement: Supplementary file 1 [file jcm-14-06265-s001.zip › jcm-3817211-supplementary materials.pdf]

Manuscript title: Geographic variation in persistence of oral anticoagulant treatment among patients with non-valvular atrial fibrillation in the United States

Supplementary Material

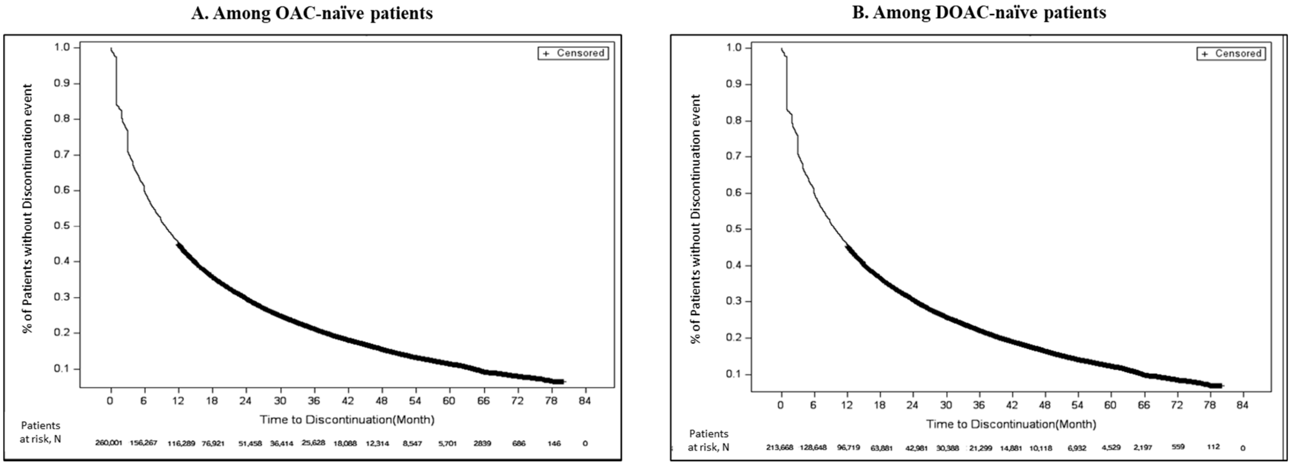

Figure S1. Kaplan-Meier survival curves for time to discontinuation (by 30-day gap) among OAC-naïve and DOAC-naïve patients – Sensitivity analysis. DOAC–direct oral anticoagulant; OAC–oral anticoagulant

Table S1. Other baseline demographic and clinical characteristics among OAC- and DOAC-naïve patients with NVAF at high risk of stroke.

Characteristic	OAC-naïve population N=260,001 (100%)	DOAC-naïve population[a] N=213,668 (100%)
**Demographic characteristics (measured as of index date)**		
Age group in years, n (%)		
18-34	439 (0.2%)	374 (0.2%)
35-49	6,667 (2.6%)	5,806 (2.7%)
50-64	58,015 (22.3%)	50,902 (23.8%)
≥65	194,880 (75.0%)	156,586 (73.3%)
Plan type, n (%)		
Fee-for-service	813 (0.3%)	599 (0.3%)
Preferred provider organization	57,807 (22.2%)	49,490 (23.2%)
Exclusive provider organization	2,792 (1.1%)	2,514 (1.2%)
Health maintenance organization	77,866 (29.9%)	64,362 (30.1%)
Point of sale with/without capitation	6,892 (2.7%)	5,836 (2.7%)
Other	36,503 (14.0%)	30,650 (14.3%)
Unknown	77,328 (29.7%)	60,217 (28.2%)
Year of index treatment, n (%)		
2016	42,492 (16.3%)	30,452 (14.3%)
2017	82,162 (31.6%)	58,511 (27.4%)
2018	38,583 (14.8%)	34,018 (15.9%)
2019	35,211 (13.5%)	32,257 (15.1%)
2020	34,457 (13.3%)	32,439 (15.2%)
2021	27,096 (10.4%)	25,991 (12.2%)
**Clinical characteristics (measured during baseline period)**		
CCI categories, n (%)		
0 (low)	35,029 (13.5%)	29,108 (13.6%)
1	49,018 (18.9%)	41,268 (19.3%)
2	47,326 (18.2%)	39,087 (18.3%)
≥3 (high)	128,628 (49.5%)	104,205 (48.8%)
CHA_2DS_2-VASc score categories, n (%)		
2	59,899 (23.0%)	52,388 (24.5%)
3	68,600 (26.4%)	56,233 (26.3%)
≥4	131,502 (50.6%)	105,047 (49.2%)
HAS-BLED score categories, n (%)		
0-1	6,131 (2.4%)	4,805 (2.2%)
2-3	128,859 (49.6%)	107,083 (50.1%)
≥4	125,011 (48.1%)	101,780 (47.6%)
Unknown	1,116 (0.1%)	969 (0.2%)
Other baseline comorbidities, n (%)		
History of bleeding	39,111 (15.0%)	31,324 (14.7%)

History of stroke/SE	65,305 (25.1%)	53,601 (25.1%)
Myocardial infarction	36,800 (14.2%)	30,404 (14.2%)
Peripheral artery disease	58,026 (22.3%)	46,990 (22.0%)
Dyspepsia or stomach discomfort	47,836 (18.4%)	40,579 (19.0%)
Renal disease	75,188 (28.9%)	60,323 (28.2%)
Liver disease	19,049 (7.3%)	16,341 (7.6%)
Non-stroke/SE peripheral vascular disease	57,364 (22.1%)	46,497 (21.8%)
Transient ischemic attack	31,309 (12.0%)	26,047 (12.2%)
Baseline medications use, n (%)		
ACE inhibitor	78,786 (30.3%)	66,930 (31.3%)
ARB	61,751 (23.8%)	54,002 (25.3%)
Calcium channel blocker	91,105 (35.0%)	78,224 (36.6%)
Inducer of warfarin	78,781 (30.3%)	67,632 (31.7%)
Proton pump inhibitor	66,309 (25.5%)	57,109 (26.7%)
Antiplatelet	43,069 (16.6%)	37,637 (17.6%)
Glucocorticoid (oral and injectable)	73,933 (28.4%)	63,560 (29.7%)
Metformin	48,928 (18.8%)	42,557 (19.9%)
Antiulcer agent	73,052 (28.1%)	62,923 (29.4%)
Antidepressant	60,531 (23.3%)	52,286 (24.5%)

ACE–angiotensin-converting enzyme; ARB–angiotensin receptor blocker; CCI–Charlson Comorbidity index; DOAC–direct oral anticoagulant; NVAF–nonvalvular atrial fibrillation; OAC–oral anticoagulant; Q1–25th percentile; Q3–75th percentile; SD–standard deviation; SE–systemic embolism.

[a]The DOAC-naïve population with NVAF represents a subcohort of the OAC-naïve population with NVAF.

Table S2. Outcomes for OAC- and DOAC-naïve patients with NVAF at time of follow up after reducing treatment gap from ≥60 to ≥30 days to define discontinuation (sensitivity analyses).

Characteristic	OAC-naïve population	DOAC-naïve population
**Discontinuation during entire follow-up period**		
Proportion of discontinuers, n (%)	199,495 (76.7%)	161,281 (75.5%)
Time to discontinuation with 30-day gap, days		
Mean (SD)	302 (357)	297 (355)
Median (Q1–Q3)	174 (59–403)	165 (59–396)
**Time to discontinuation during 12-month follow-up period**		
Proportion of discontinuers, n (%)	137,650 (52.9%)	112,119 (52.5%)
Time to discontinuation with 30-day gap, days		
Mean (SD)	115 (90)	113 (90)
Median (Q1–Q3)	89 (29–179)	89 (29–179)
**Persistence among patients with up to 12 months of follow up**		
Proportion of patients, n (%)		
Persistent at 6 months	156,267 (60.1%)	128,648 (60.2%)
Persistent at 9 months	133,728 (51.4%)	110,637 (51.8%)
Persistent at 12 months	122,351 (47.1%)	101,549 (47.5%)
**Persistence among subset of patients with up to 18 months of follow up**		
Proportion of patients, n (%)		
Persistent at 18 months[a]	81,974 (35.8%)	68,204 (36.2%)

DOAC–direct oral anticoagulant; NVAF–nonvalvular atrial fibrillation; OAC–oral anticoagulant; Q1–25th percentile; Q3–75th percentile; SD–standard deviation.

[a] The DOAC-naïve population with NVAF represents a subcohort of the OAC-naïve population with NVAF.

Table S3. Mean number of claims for all OAC-naïve and DOAC-naïve patients with NVAF by region and days' supply.

Cohort	Region	Total study population (N)	30-day supply		90-day supply	
			Number of Claims	Average claims per patient	Number of Claims	Average claims per patient
All OAC-naïve patients[a]	Northeast	72,507	332,905	4.6	122,359	1.7
	Midwest	59,979	258,358	4.3	101,187	1.7
	South	83,880	377,243	4.5	124,973	1.5
	West	42,778	190,363	4.4	63,625	1.5
All DOAC-naïve patients[a]	Northeast	59,723	293,276	4.9	94,356	1.6
	Midwest	46,945	219,265	4.7	74,720	1.6
	South	71,614	337,951	4.7	100,171	1.4
	West	34,632	163,031	4.7	49,289	1.4
Persistent OAC-naïve patients[b]	Northeast	46,013	266,823	5.8	101,790	2.2
	Midwest	37,353	204,467	5.5	83,890	2.2
	South	47,930	286,579	6.0	100,952	2.1
	West	24,918	145,912	5.9	51,505	2.1
Persistent DOAC-naïve patients[b]	Northeast	38,040	236,298	6.2	79,415	2.1
	Midwest	29,124	174,076	6.0	62,709	2.2
	South	40,741	257,247	6.3	81,539	2.0
	West	19,961	124,807	6.3	40,190	2.0

DOAC–direct oral anticoagulant; NVAF–nonvalvular atrial fibrillation; OAC–oral anticoagulant.

[a] This was a claims level analysis among all OAC-naïve and DOAC-naïve patients with NVAF and >1 prescription claim (including index claims) was considered.

[b] This was a claims level analysis among all persistent OAC-naïve and DOAC-naïve patients with NVAF and >1 prescription claim (including index claims) was considered.

Note: Other and Unknown regions were excluded in the above analysis.

Table S4. Distribution of 30- and 90-days' supply among OAC-naïve and DOAC-naïve patients with NVAF with >1 prescription claims by geographic region.

Cohort	Region	Total claims (N)	30-day supply		90-day supply	
			Number of Claims	Average claims per patient	Number of Claims	Average claims per patient
OAC-naïve[a]	Northeast	458,083	289,534	63%	105,692	23%
	Midwest	363,970	224,158	62%	86,672	24%
	South	479,295	326,503	68%	106,706	22%
	West	254,399	165,409	65%	54,579	21%
	Other/Unknown	5,928	5,031	85%	707	12%
	Total	1,561,675	1,010,635	65%	354,356	23%
DOAC-naïve[a]	Northeast	378,467	254,488	67%	82,827	22%
	Midwest	284,403	189,982	67%	64,887	23%
	South	412,089	292,036	71%	86,623	21%
	West	204,217	141,267	69%	42,834	21%
	Other/Unknown	5,185	4,438	86%	639	12%
	Total	1,284,361	882,211	69%	277,810	22%

Key: OAC–oral anticoagulant; NVAF–non-valvular atrial fibrillation; Other/Unknown includes Puerto Rico, Guam, Virgin Islands, and missing or unknown region.

[a] The above claims analysis among all OAC-naïve and DOAC-naïve patients excluded index claims.
